# Use of DNA melting simulation software for *in silico *diagnostic assay design: targeting regions with complex melting curves and confirmation by real-time PCR using intercalating dyes

**DOI:** 10.1186/1471-2105-8-107

**Published:** 2007-03-29

**Authors:** John P Rasmussen, Christopher P Saint, Paul T Monis

**Affiliations:** 1 Cooperative Research Centre for Water Quality and Treatment, Australian Water Quality Centre, SA Water Corporation, Salisbury, SA, 5108 Australia; 2 School of Pharmacy and Medical Sciences, University of South Australia, City East Campus, Adelaide, SA, 5000 Australia

## Abstract

**Background:**

DNA melting curve analysis using double-stranded DNA-specific dyes such as SYTO9 produce complex and reproducible melting profiles, resulting in the detection of multiple melting peaks from a single amplicon and allowing the discrimination of different species. We compare the melting curves of several *Naegleria *and *Cryptosporidium *amplicons generated *in vitro *with *in silico *DNA melting simulations using the programs POLAND and MELTSIM., then test the utility of these programs for assay design using a genetic marker for toxin production in cyanobacteria.

**Results:**

The SYTO9 melting curve profiles of three species of *Naegleria *and two species of *Cryptosporidium *were similar to POLAND and MELTSIM melting simulations, excepting some differences in the relative peak heights and the absolute melting temperatures of these peaks. MELTSIM and POLAND were used to screen sequences from a putative toxin gene in two different species of cyanobacteria and identify regions exhibiting diagnostic melting profiles. For one of these diagnostic regions the POLAND and MELTSIM melting simulations were observed to be different, with POLAND more accurately predicting the melting curve generated *in vitro*. Upon further investigation of this region with MELTSIM, inconsistencies between the melting simulation for forward and reverse complement sequences were observed. The assay was used to accurately type twenty seven cyanobacterial DNA extracts *in vitro*.

**Conclusion:**

Whilst neither POLAND nor MELTSIM simulation programs were capable of exactly predicting DNA dissociation in the presence of an intercalating dye, the programs were successfully used as tools to identify regions where melting curve differences could be exploited for diagnostic melting curve assay design. Refinements in the simulation parameters would be required to account for the effect of the intercalating dye and salt concentrations used in real-time PCR. The agreement between the melting curve simulations for different species of *Naegleria *and *Cryptosporidium *and the complex melting profiles generated *in vitro *using SYTO9 verified that the complex melting profile of PCR amplicons was solely the result of DNA dissociation. Other data outputs from these simulations were also used to identify the melting domains that contributed to the observed melting peaks for each of the different PCR amplicons.

## Background

Differentiation of PCR products using DNA melting curve analysis was first demonstrated by Ririe *et al *[1] with the double-stranded DNA-specific dye SYBR Green I and has since seen widespread adoption in real-time PCR applications [2]. Melting curve analysis provides immediate practical benefits in real-time PCR, obviating the need for gel electrophoresis by providing a reproducible signature of the amplified DNA sequence that may be used for typing PCR products [1]. Typing is typically achieved by examining the first derivative of the melting curve and identifying the characteristic "melt peak" (*T*_m_), which is the temperature at which the rate of fluorescence change (DNA denaturation) is highest and is observed in the raw data as a sudden decrease in fluorescence [1].

Relatively few melting curve assays are based on the melting profile of the entire PCR product, with many measuring the melting of specific probes from the region of interest, typically targeting single nucleotide polymorphisms (eg. fluorescent resonance energy transfer (FRET) probes [3]). Whole product melting curve analysis with SYBR Green I has been used to a lesser degree in diagnostic assays for cancer treatment monitoring [4] and pathogens [5-9]. Almost without exception, these assays produce simple melting curves which result in a single peak [4-9]; however, melting curve analysis need not be limited to product differentiation on the basis of a single peak and associated *T*_m_. Wittwer *et al *[10] identified two melting domains in a 550 bp amplicon of the hydroxytryptamine receptor 2A gene and amplicons with multiple melting domains have been used to differentiate species of *Giardia *(660 bp amplicon from the *gdh *gene) [11] and *Naegleria *(350 – 400 bp amplicons from the intergenic spacer region) [12].

The idea that large double-stranded DNA fragments of several kilobases may contain several melting domains is not new [13], and was pursued for many years by Poland [14] and others [13-22]. This body of work has provided several important insights into the process of DNA melting: that it is co-operative [13,14]; that it is both sequence and nucleotide position dependent [20]; and that it is also subject to smaller local effects [21,22]. The possibility that multiple melting domains existed in DNA fragments less than a single kilobase does not appear to have been examined in these studies.

Two publicly available bioinformatic resources have been developed to model DNA melting: POLAND [23] and MELTSIM [24]. We evaluate the ability of these programs to simulate melting curves for the intergenic spacer region from three species of the waterborne protozoan *Naegleria *and a segment of 18S rDNA from two species of *Cryptosporidium*. We also describe the use of these resources to design an informative melting curve assay for two different species of toxic cyanobacteria *in silico *and then physically test the assay in the laboratory to confirm assay performance with real samples.

## Results

### Comparison of simulated and experimental melting curves for protozoa

**Figure 1:**
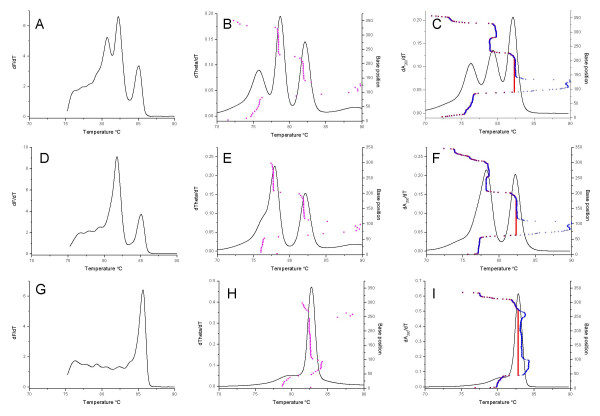
**Comparison of real-time PCR and simulated melting profiles for different *Naegleria *species**. Plots (solid lines) of the first derivatives (for either fluorescence, theta or absorbance) versus temperature for *Naegleria fowleri *(A, B, C), *Naegleria lovaniensis *(D, E, F) and *Naegleria australiensis *(G, H, I) for either DNA amplified and melted in the presence of SYTO9 (A, D, G) or melting simulations conducted using the amplicon DNA sequences and either MELTSIM (B, E, H) or POLAND (C, F, I) respectively. The melt map for each sequence (base position versus predicted *T*_m_) determined by MELTSIM is indicated by magenta circles. The equivalent profiles calculated by POLAND are indicated by blue plus signs or red squares for first order and second order reactions respectively.

The SYTO9 melting curves of amplicons from the intergenic spacer region of *N. australiensis*, *N. fowleri *and *N. lovaniensis *were very distinct as previously reported [12], with multiple peaks differing in shape and height: the *N. australiensis *amplicon melted with a single sharp peak (Fig 1G); the *N. fowleri *amplicon melted with three peaks all at different heights (Fig 1A); and the *N. lovaniensis *amplicon melted with two peaks, the first approximately double the amplitude of the second (Figure 1D). When the sequence of each *Naegleria *amplicon was subjected to a melting simulation using the POLAND (Figure 1C, F, I) and MELTSIM (Figure 1B, E, H) programs, the predicted melting curves were similar to the profiles obtained using SYTO9. The number of melt peaks and the separation of these peaks were consistent between the simulation programs and the physical data resulting from real-time PCR melting curve analysis. The The predicted relative peak heights (height of a given peak relative to the other peaks for a given template) for *N. fowleri *and *N. lovaniensis *did not exactly match the physical data (Figure 1A, D), although the MELTSIM profiles (Figure 1B, E) appeared to be a closer prediction compared to POLAND (Figure 1C, F). The values of the *T*_m_s predicted by either program did not match the *T*_m_s obtained by DNA melting curve analysis using SYTO9. The melting maps (*T*_m _predicted for each base position (or domain) versus base position) were similar for the POLAND first order reaction (Fig 1C, F) and MELTSIM (Figure 1B, E) predictions for *N. fowleri *and *N. lovaniensis*, but slightly different for *N. australiensis*, particularly in the region at around 250 bp. In the case of MELTSIM, the melting map did not cover the entire amplicon and appeared to be truncated at 357 bp for *N. fowleri *and 298 bp for *N. lovaniensis *and *N. australiensis*.

**Figure 2:**
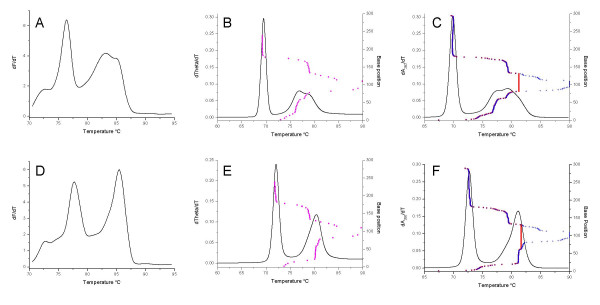
**Comparison of real-time PCR and simulated melting profiles for different *Cryptosporidium *species**. Plots (solid lines) of the first derivatives (for either fluorescence, theta or absorbance) versus temperature for *Cryptosporidium parvum *(A, B, C) and *Cryptosporidium muris *(D, E, F) for either DNA amplified and melted in the presence of SYTO9 (A, D) or melting simulations conducted using the amplicon DNA sequences and either MELTSIM (B, E) or POLAND (C, F) respectively. The melt map for each sequence (base position versus predicted *T*_m_) determined by MELTSIM is indicated by magenta circles. The equivalent profiles calculated by POLAND are indicated by blue plus signs or red squares for first order and second order reactions respectively.

A segment of 18S rDNA from *Cryptosporidium parvum *and *Cryptosporidium muris*, approximately 300 bp, was amplified and melted in the presence of SYTO9. The differentiated melting curves resulted in complex profiles that allowed the ready differentiation of these two species, with *C. muris *producing 2 peaks with *T*_m_s of 75.5°C and 83.5°C (Figure 2D) and *C. parvum *producing a peak at 73.8°C and a peak with a shoulder at 80.8°C and 83.0°C respectively (Figure 2A). The standard deviation in *T*_m _observed between replicates (n = 4 for each species) was less than 0.3°C (data not shown). In the case of the *C. parvum *sequence there was a subtle difference between the POLAND (Figure 2C) and MELTSIM (Figure 2B) predictions, with a difference between the position and height of the second peak and its shoulder. The MELTSIM profile more closely matched the observed profile (Figure 2A). The melting profiles predicted *C. muris *by MELTSIM (Figure 2E) and POLAND (Figure 2F) were the same as each other and similar to the observed profile (Figure 2D) in terms of the number of peaks and spacing between peaks. In both cases the peak heights and absolute values *T*_m_s differed between the *in vitro *and *in silico *profiles. The melt maps predicted by each program were similar to each other for *C. parvum *(Figure 2B, 2C) and *C. muris *(Figure 2E, F); however, the MELTSIM maps were again truncated, here at 238 bp.

**Figure 3:**
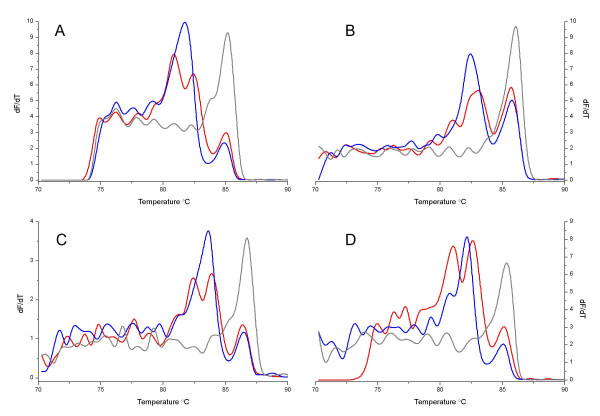
**Comparison of melting profiles obtained using different intercalating dyes and purified *Naegleria *amplicons**. First derivative plots for purified amplified DNA from *Naegleria fowleri *(red line), *Naegleria lovaniensis *(blue line) and *Naegleria australiensis *(black line) melted in the presence of either SYTO9 (A), SYBR Green (B), LC Green I (C) or SYTO59 (D).

To establish that the observed similarity in predicted and actual meting curves was not a "dye-specific" phenomenon, *N. australiensis*, *N. fowleri *and *N. lovaniensis *amplicons were purified and added in equal amounts (ng) to a solution containing PCR buffer, MgCl_2 _and one of four different double-strand DNA-specific binding dyes: SYTO9 [11], SYBR Green I [1], LC Green [10] and SYTO59 (Monis and Rasmussen *unpublished*). Whilst each of the dyes was used at an optimum concentration, analysing the melting curves under otherwise standard conditions limited any variability that may be contributed by differing reaction conditions or PCR amplification. The comparison of melting curves for each of the *Naegleria *test amplicons using the four dyes (Figure 3A–D) with melting curve simulations generated by POLAND and MELTSIM showed good agreement in the number of peaks and the peak separation for each of the dyes. The shape of the peaks and peak height were somewhat variable when different dyes were compared, qualitatively most different in the SYBR Green I melting curve (Figure 3B).

When comparing the melting curves for purified *Naegleria *amplicons with post-purification SYTO9 addition (Figure 3A) and for *Naegleria *template amplified in the presence of SYTO9 (Figure 1A) the number of peaks, peak shape and peak separation were similar. Peak heights were also similar with the exception of the first and second peaks in the melting curve for the *N. fowleri *amplicon. The *T*_m _of the peaks differed only slightly.

### Use of simulation software to identify diagnostic profiles for delineation of cyanobacterial species and comparison with experimental melting curves

**Figure 4:**
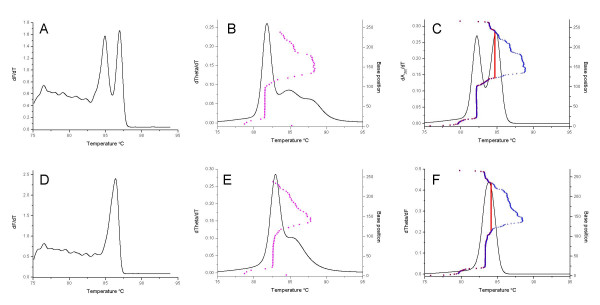
**Comparison of real-time PCR and simulated melting profiles for different cyanobacterial species**. Plots (solid lines) of the first derivatives (for either fluorescence, theta or absorbance) versus temperature for *Cylindrospermopsis raciborskii *(A, B, C) and *Aphanizomenon ovalisporum *(D, E, F) for either DNA amplified and melted in the presence of SYTO9 (A, D) or melting simulations conducted using the amplicon DNA sequences and either MELTSIM (B, E) or POLAND (C, F) respectively. The melt map for each sequence (base position versus predicted *T*_m_) determined by MELTSIM is indicated by magenta circles. The equivalent profiles calculated by POLAND are indicated by blue plus signs or red squares for first order and second order reactions respectively.

To test the utility of the melting simulation programs for the design of diagnostic melting curve assays, POLAND and MELTSIM were used to screen the DNA sequences of the *cynD*/*AoaB *gene, a part of the gene cluster implicated in the production of the toxin cylindrospermopsin in the cyanobacteria *Cylindrospermopsis raciborskii *[25] and *Aphanizomenon ovalisporum *[26], respectively. In screening the *cynD*/*AoaB *gene sequences, a 267 bp segment that was predicted to differentiate the two species was identified and resulted in different melting curve simulations for POLAND (Figure 4C, F) and MELTSIM (Figure 4B, E). The POLAND algorithm generated two peaks for the *C. raciborskii *sequence (Figure 4C) and one for the *A. ovalisporum *sequence (Figure 4F), whilst the MELTSIM program generated one peak with a shoulder on the right hand side for *A. ovalisporum *(Figure 4E) and a single major peak with a minor peak and extended shoulder on the right hand side for *C. raciborskii *(Figure 4B). As observed with the previous simulations the melt maps generated by MELTSIM and POLAND were similar but the MELTSIM map appeared to stop at 238 bp.

Flanking primers were designed for the 5' and 3' ends of this segment, where the sequences were identical between the two different cyanobacterial species. The primers were used to amplify template DNA from these species in the presence of SYTO9, and the resulting 267 bp amplicons were analyzed by melting curve analysis (Figure 4A, D), producing profiles matching the POLAND melting simulations in terms of the number and separation of melt peaks and relative peak heights. The values of the *T*_m_s predicted by either program did not match the *T*_m_s obtained by DNA melting curve analysis using SYTO9.

The SYTO9 melting curve assay for the *cynD*/*AoaB *gene was used to type a set of 27 cylindrospermopsin-producing and non-toxin producing cyanobacterial DNA extracts (Table 1). A total of 17 of the 27 extracts contained the putative toxin gene and were rapidly genotyped into two groups on the basis of the melting curve analysis.

**Table 1:** Typing of cyanobacterial strains using the *cynD*/*AoaB *SYTO9 melting curve assay.

**Species**	**Strain**	**Peak 1 *T*_m _°C**	**Peak 2 *T*_m _°C**	**Toxic**
*C. raciborskii*	AWT205	84.9	87.0	yes
*C. raciborskii*	CYLI-19	-	-	no
*C. raciborskii*	CYLI-29	-	-	no
*C. raciborskii*	CYLI-31	-	-	no
*C. raciborskii*	CYLI-42	-	-	no
*C. raciborskii*	CYLI-53	-	-	no
*C. raciborskii*	CYLI-75	-	-	no
*C. raciborskii*	CYP003A	85.0	87.0	yes
*C. raciborskii*	CYP005F	85.0	87.0	yes
*C. raciborskii*	CYP009A	84.9	86.8	yes
*C. raciborskii*	CYP010A	84.9	86.8	yes
*C. raciborskii*	CYP014A	-	-	no
*C. raciborskii*	CYP023A	85.0	87.0	yes
*C. raciborskii*	CYP024C	85.0	87.0	yes
*C. raciborskii*	CYP025B	85.0	87.0	yes
*C. raciborskii*	CYP026J	85.0	87.0	yes
*C. raciborskii*	CYP030	85.0	87.0	yes
*C. raciborskii*	CYP033	84.9	87.0	yes
*A. bergii*	ANA360D	-	-	no
*A. bergii*	ANA360H	-	-	no
*A. bergii*	ANA366A	86.3	-	yes
*A. bergii*	ANA283A	86.6	-	yes
*A. ovalisporum*	APH028A	-	-	no
*A. ovalisporum*	APH031G	86.4	-	yes
*A. ovalisporum*	APH033C	86.4	-	yes
*A. ovalisporum*	APH035E	86.4	-	yes
*A. ovalisporum*	APH035F	86.4	-	yes

Typing of cylindrospermopsin-producing and non-producing cyanobacterial strains using a SYTO9 melting curve assay for the *cynD/AoaB *gene designed *in silico *using the POLAND software. The *T*_m _for each melt peak observed in *cynD/AoaB *positive samples is reported to one decimal place. All *cynD/AoaB *negative samples are indicated by "-". Toxin production confirmed by LC-MS with a detection limit of 0.2 μg/L ([34]).

## Discussion

Complex melting profiles resulting in multiple melting peaks provide a superior tool for identification of species or characterization of amplicons compared with simple profiles that result in only a single peak. In addition to *T*_m_, both the number of peaks and relative peak heights can be used as additional diagnostic characters to facilitate identification. Until this study, a systematic method for developing such assays had not been reported. The ability to simply submit a DNA sequence to melting simulation software and generate a useful prediction of the likely real-time PCR melting curve profile *in silico *was an interesting possibility that we firstly explored using existing SYTO9 melting curve assays that resulted in complex melt profiles. Melting curves for the intergenic spacer region from each of the three *Naegleria *species had clearly distinct profiles (*N. australiensis *one peak; *N. lovaniensis *two peaks; and *N. fowleri *three peaks) and in each case the number of peaks and the separation of the peaks were predicted by the POLAND [23] and MELTSIM [24] programs, with the MELTSIM simulations providing a more accurate predictor of relative peak heights for the *Naegleria *amplicons. Similarly, melting curves of a segment of 18S rDNA from *C. parvum *and *C. muris *resulted in distinct profiles that were predicted by the simulation programs, with MELTSIM providing a better prediction of the *C. parvum *melting curve. In both cases the actual *T*_m_s determined using SYTO9 melting curve analysis were generally 3 – 5°C higher than the predicted *T*_m_s.

The difference observed between the experimental and predicted *T*_m_s is expected considering that the thermodynamic values used in the simulations for base pair stabilities or base stacking are based on empirical data determined for specific salt concentrations in the absence of any intercalating dyes (eg. Blake and Delcourt [20]). Considering the nature of the interaction between intercalating dyes and dsDNA [27] it would be expected that intercalating dyes are likely to increase the *T*_m_, and this is what is observed for the experimental data compared with the melting simulations. While it would be useful for the simulations to more accurately predict the *T*_m _under real-time PCR conditions (for the programs to be used as a assay design tools), in practice this will be difficult considering that different dyes might interact with DNA differently and that the interaction might be affected by the ratio of dye:DNA molecules.

The close agreement in the *Naegleria *SYTO9 melting curve profiles and melting simulations generated using POLAND or MELTSIM was not limited to the use of SYTO9. Three other double-stranded DNA-specific dyes were combined with purified *Naegleria *test amplicons, in addition to SYTO9; and resulted in melting curves with the same number of peaks and similar peak separation to simulations generated by POLAND and MELTSIM. Between-dye comparisons demonstrated that relative peak height varied slightly between dyes, most obviously for the *N. fowleri *melting curve, whilst the shape of the peaks was most obviously different when SYBR Green I was used. The melt map of the *N. fowleri *sequence suggests that the first 75 base-pairs of the sequence represent the melt domain contributing to the first peak. This region is AT-rich (73% A+T) which, considering the GC-preference of SYBR Green I [10,28], may explain the loss of this peak for the SYBR Green I melting curve.

To test the utility of the POLAND and MELTSIM programs for *in silico *melt curve assay design we chose a target gene of more than 6 Kb, *cynD*/*AoaB*, implicated in the biosynthesis of the cyanotoxin cylindrospermopsin [25,29]. The program interface for both melt simulation algorithms permits submission and analysis of only one sequence at a time. This limitation meant that sequences could only be screened by visually identifying regions of sequence polymorphism in a multiple alignment of the *cynD*/*AoaB *gene homologues, then manually submitting individual polymorphic regions to melt simulation. Screening sequences firstly with POLAND, we identified a 267 bp sequence segment that generated distinctly different melt profiles depending on the origin of the sequence: two melt peaks for *cynD *from *C. raciborskii *and one peak for *AoaB *from *A. ovalisporum*. When these two sequences were submitted to MELTSIM the simulation likewise predicted one melt peak for *AoaB *sequence; however, only one melt peak was predicted for *cynD *sequence, although the *T*_m _was different to the *AoaB *sequence and there was a sub-peak/shoulder that was not present in the *AoaB *profile. The substantial differences in the simulated melting curves for the *cynD *sequence were surprising when compared to the melting simulations for the *Naegleria *and *Cryptosporidium *amplicons, where POLAND and MELTSIM predictions were similar except for fine scale changes. Comparison between these simulations and actual SYTO9 melting curve analysis of the *cynD *amplicon leaves no doubt that the POLAND program is clearly better enabled to model the melting in this case.

The underlying basis for the differences observed between MELTSIM and POLAND are likely embedded in the way the two programs implement the Poland algorithm and incorporate the thermodynamic parameters used for base stacking, loop opening and closing and so on. A difference between MELTSIM and POLAND is the base pair stability parameters that they use, with POLAND using the values determined by Blake and Delcourt in 1998 [20], whereas the Windows release of MELTSIM uses parameters determined by the authors of the software in 1995. Preliminary comparisons using Linux compilations of MELTSIM [30] incorporating either the 1995 or 1998 stability parameters suggest that there is little difference between predictions using either set of parameters on *cynD *and *AoaB *sequences, with the same major peak identified and only minor differences in other regions of the profile (data not shown).

Examination of the melt maps generated by MELTSIM show that the predicted melt peaks correlate with sequence domains comprising runs of 50 or more base pairs with the same or similar *T*_m_, resulting in a peak with a height proportional to the relative size of the domain. In contrast, the POLAND melt peaks appear to correspond with domains identified from the melt map generated using second order reaction probabilities. This causes POLAND to overestimate peak heights in some instances (eg Figure 1C, 1F, 2C) but ironically also appears to be why POLAND was able to predict two peaks for the *cynD *amplicon.

Interestingly, the melt profile of *C. parvum *has a similar overall shape to the predicted profile for *cynD*. A difference between these two sequences is that the lower *T*_m _domain in *C. parvum *is at the 3' end whereas it is at the 5' end in *cynD*. Analysis of the reverse complement of *cynD *(such that the melt map would be more comparable with *C. parvum*) yielded a different melt profile to the original with a much more obvious second peak half the height of the first peak (data not shown), more similar to the observed profile. This finding is surprising, especially considering that the melt map of the reverse complement appeared to be similar to the original except for the orientation and for the fact that the entire reverse complemented 3' end was included in the melt map (it was truncated in the original analysis because the MELTSIM melt map stopped after 238 bp). This raises the possibility that some data might be missing from the MELTSIM simulation, resulting in the loss of part of the domain and the subsequent failure to detect the second peak. Analysis of the reverse complementary sequences of *C. parvum *and *N. fowleri *resulted in no change to the profiles, although the *T*_m _of the main *C. parvum *peak shifted slightly to the right by approximately 1°C (data not shown).

The truncation of the MELTSIM melt map appears to be caused by rounding during the calculation of domain sizes. The maximum number of domains per block (sequence) is 60, and it appears that MELTSIM calculates block size by dividing the sequence length by the number of domains per block and rounding the resulting number down to the nearest whole number. The melt map only allows plotting of 60 domains, which in the case of the sequences used in this study meant that sequences were truncated at approximately 240, 300 or 360 bp. Preliminary experiments conducted using a Linux compilation of MELTSIM [30], modified to use the 1995 parameters and to allow >60 domains, allowed the melt map to be corrected by setting the number of domains to be one less than the sequence length (ie domain size = 1 bp), providing the melt map for the entire sequence (data not shown). This modification did not appear to have any effect on the predicted melting profile for *cynB*, suggesting that MELTSIM does not use the melt map output directly in the prediction of the melting curve.

The differences in the melting curves for the *cynD*/*AoaB *amplicons, initially identified by melting curve simulation, were exploited in a diagnostic assay to type known strains of cyanobacteria that produce the cyanotoxin cylindrospermopsin. Actual amplification of the expected 267 bp amplicon indicated that the strain was potentially toxic and the melting curve analysis confirmed that the correct product had been amplified and also typed the strain according to its genus. The *T*_m _for each of the melt peaks observed was very uniform and divided the strains into two distinct groups. The melting curve analyses for *C. raciborskii *and *A. ovalisporum *were completely concordant with taxonomic classification. Notably, two *Anabaena bergii *strains were observed to bear sequences similar to the *AoaB *gene on the basis of the SYTO9 melting curve analysis. Later phylogenetic investigation of these strains using several highly conserved sequences confirmed that these were initially misidentified and were actually *A. ovalisporum *(data not shown). Whilst the assay proved to be highly informative for isolated cyanobacterial strains, it may also be used for routine testing of environmental samples, since cases of algal blooms that contain more than one species of cylindrospermopsin-producing cyanobacteria are rare (Dr. Peter Baker *pers*. *comm*.). The possibility also remains open that single filaments of cyanobacteria may be taken from a live environmental sample, extracted and the DNA amplified by real-time PCR.

Sampling extant sequence polymorphism by melting curve analysis of whole products and using the melting curve "signatures" for biotyping may be less discrete than melting curve analysis of single nucleotide polymorphisms (SNP) (e.g. using oligonucleotides for FRET). However, for some applications, such as species identification, the use of whole product melting curve analysis may be preferred because it is more tolerant to any small sequence differences that might result due to intraspecific variation. In addition, identification using whole product melting curve profiles is likely to be more robust than a SNP because by its nature a SNP is relying on only a single character difference for identification, whereas multiple character differences are usually required to change the melting curve profile of a larger fragment. The POLAND or MELTSIM programs will in their present form be useful as a guide to melt curve assay design but with some further refinement might be adapted into dedicated melting curve assay design software that provides access to the full richness of informative sequence polymorphism already present in sequence databases.

## Conclusion

Multiple melting domains in a melting curve provide a rich source of information for typing samples. Computer-aided melting simulation provides a gateway to the exploitation of this information. The demonstration that melting curve assays can be designed reliably *in silico *may stimulate more widespread use of whole product melting curve analysis and ultimately lead to dedicated melting curve assay design software.

## Methods

### Microbial strains and culturing

The cyanobacteria *C. raciborskii *and *A. ovalisporum *were cultured as described by Wilson *et al *[29] and the protozoa *N. australiensis*, *N. fowleri *and *N. lovaniensis *were prepared by aseptic transfer of a single plaque to a 1.5% non-nutrient agar plate (Oxoid, Adelaide, Australia) supplemented with 300 μL *E. coli *(3 × 10^10 ^cells/ml) in sterile saline. All subcultures were derived from the Australian Water Quality Centre Culture collection. *C. parvum *oocysts (Swiss cattle C26) were purchased from the Department of Veterinary and Biomedical Sciences, Murdoch University, Perth, Australia. *C. muris *oocysts were kindly provided by Dr Una Ryan.

### DNA extraction

For cyanobacterial cultures, sub-samples of 1 ml were taken from densely grown cultures and pelleted by centrifugation at 14000 RPM for 10 min in a Sigma 1–15 Microfuge (Sigma, Germany). The supernatant was removed by sterile pipetting and DNA extracted from the remaining pellet using the QIAamp DNA Minikit (Qiagen, Germany) according to the Tissue Extraction protocol supplied by the manufacturer. For *Naegleria *cultures, a loopful of cells was aseptically transferred from the edge of a plaque into 20 μL of Instagene (Bio-Rad USA) and DNA extracted from the cells according to the protocol supplied by the manufacturer. Oocyst DNA was extracted by 5 cycles of freeze thawing in liquid nitrogen followed by Instagene extraction following the manufacturer's instructions.

### Amplification and melting curve analysis in the presence of SYTO9

PCRs were performed using a Rotor-Gene 3000 (Corbett Research, Sydney, Australia). *Cryptosporidium *18S rDNA was amplified as described by Keegan *et al *[31], except that the Taqman probe was excluded and SYTO9 was added at a final concentration of 2.5 μM. *Naegleria *PCR was performed as described by Robinson *et al *[12]. Cyanobacterial PCRs were performed in 25 μL reaction volumes containing variant amounts of genomic DNA, 500 nM of forward and reverse primer (Geneworks, Australia), 0.2 μM deoxynucleotide triphosphates (Promega, USA), 1 × Platinum *Taq *PCR buffer (Invitrogen, USA), 1 U of Platinum *Taq *(Invitrogen, USA), 2.5 μM SYTO9 and 4 mM MgCl_2 _(Invitrogen, USA). For cyanobacterial templates, the thermal cycling conditions included an initial denaturation at 94°C for 2 min, followed by 50 cycles at 94°C for 5 s and 60°C for 25 s, with data acquisition on the FAM channel following the 60°C anneal/extension step. The amplified PCR products were visualised on 1.5% agarose gels stained with Gelstar (Cambrex Bio Science, USA) to check product formation and sizes of the products were estimated from a DNA molecular weight marker: pUC19/HpaII digest (Geneworks, Australia).

Melting curve conditions included an initial hold step of 70°C for 1 min, followed by temperature ramping of 0.5°C/30 s to 99°C. SYTO9 was detected in the FAM channel (excitation at 470 nm, detection at 510 nm) using a gain setting of 2. Melting curves were initially visualised using the Rotor Gene Analysis software (version 6) with the digital filter set to none. For preparation of graphs data were exported from Rotor Gene experiment files into Origin 7 (OriginLab Corporation) and lines were drawn through points using a b-spline.

### Melting curve analysis of *Naegleria *DNA amplified in the absence of DNA binding dye

*Naegleria *DNA was amplified as described above except that SYTO9 was omitted from the reaction mixture. Amplicons were purified using Montage PCR purification columns (Millipore, USA) according to the protocol supplied by the manufacturer. Purified amplicons amplified from the same template were pooled and the mass of DNA in each of the pools calculated by UV spectrophotometric analysis using a GeneQuant Pro (Biochrom, UK). The mass of DNA in each of the pools was adjusted to 30 ng/μL. For the melting analysis, all reactions were melted with 25 μL total reaction volumes containing 120 ng of test amplicon DNA, 2.5 mM MgCl_2 _(Perkin-Elmer, USA), 1 × PCR buffer II (Perkin-Elmer, USA) and either 0.25 × SYBR Green I (Invitrogen, USA), 1 × LC Green (IT Biochem, USA), 2 μM SYTO 9 (Invitrogen, USA) or 2 μM SYTO 59 (Invitrogen, USA). Melting curve conditions included three repetitions of the following cycle: two initial hold steps for 1 min each, 95°C then 60°C, followed by temperature ramping of 0.5°C/30 s from 70 to 99°C. SYTO9 and LC Green were detected in the FAM channel (excitation at 470 nm, detection at 510 nm) at a gain of 6 and 10 respectively, SYBR Green I was detected in the SYBR channel (excitation at 470 nm, detection at 585 nm) at a gain of 7.33 and SYTO59 was detected in the Cy5 channel (excitation at 625 nm, detection at 660 nm) using a gain setting of 8.67. Melting curves were visualised by the analysis software (Rotor Gene Analysis Software 6.0 build 34) with the digital filter set to none. The purified amplicons used in the melting analyses were visualised on 1.5% agarose gels as described above to confirm that the amplicons were single products of the correct sizes.

### Melting simulations

GenBank accession numbers for DNA sequences used in the computer simulations are: AJ132027 (*Naegleria fowleri *NG166), AJ132034 (*Naegleria australiensis *LSR34), X96569 (*Naegleria lovaniensis *F9, which is identical to *Naegleria lovaniensis *NG034), AF093496 (*Cryptosporidium muris*), AF040725 (*Cryptosporidium parvum*), AF395828 (*Aphanizomenon ovalisporum*, nucleotides 3975 – 4242) and DQ531847 (*Cylindrospermopsis raciborskii*). Where required, sequences were truncated to remove sequences outside of the region bounded by the primers. Melting simulations were performed using the POLAND program [32] and the MELTSIM for Windows program [33]. Poland was set to use the Blake and Delcourt model for melting [20], and both programs were set to simulate melting curves between 60°C and 90°C, incrementing by 0.5°C, with a fixed salt concentration of 75 mM NaCl. Critical output data relevant to this study were the "differentiated hypochromicity at 260 and 282 nm (dA/dT) *vs*. temperature" plot and the first and second order reaction stack indices for temperature pf 50% probability for POLAND and the "theta (the calculated fraction of base pairs remaining in a helical state) *vs*. temperature" plot and melt map plot for MELTSIM.

## Authors' contributions

JPR participated in experimental design, developed the cyanobacterial PCR assay, conducted the melting curve analyses and drafted the manuscript. CPS contributed to the cyanobacterial component of the work. PTM participated in experimental design, conducted the melting simulations and data analysis, prepared the figures and drafted the manuscript. All authors have read and approved the final manuscript.
